# The Oral Microbiome of King Richard III of England

**DOI:** 10.1002/ajpa.70350

**Published:** 2026-09-03

**Authors:** Irina M. Velsko, Alexander Hübner, Zandra Fagernäs, James A. Fellows Yates, Allison E. Mann, Courtney A. Hofman, Andrew T. Ozga, Cecil M. Lewis, Camilla Speller, Sarah Fiddyment, Michael Francken, Joachim Wahl, Johannes Krause, Anita Radini, Turi King, Christina Warinner

**Affiliations:** ^1^ Department of Archaeogenetics Max Planck Institute for Evolutionary Anthropology Leipzig Germany; ^2^ Archaeogenetics Research Unit Leibniz Institute for Natural Products Research and Infection Biology Hans Knöll Institute Jena Germany; ^3^ Globe Institute University of Copenhagen Copenhagen Denmark; ^4^ Department of Anthropology University of Wyoming Wyoming Laramie USA; ^5^ Department of Anthropology University of Oklahoma Norman Oklahoma USA; ^6^ Laboratories of Molecular Anthropology and Microbiome Research University of Oklahoma Norman Oklahoma USA; ^7^ Department of Biological Sciences Nova Southeastern University Fort Lauderdale Florida USA; ^8^ Department of Anthropology University of British Columbia Vancouver British Columbia Canada; ^9^ Department of Archaeology University of York York UK; ^10^ State Office for Cultural Heritage Baden‐Württemberg Konstanz Germany; ^11^ Institute for Archaeological Sciences, Section Paleoanthropology University of Tuebingen Tübingen Germany; ^12^ School of Archaeology University College Dublin Dublin Ireland; ^13^ Genetics and Genome Biology University of Leicester Leicester UK; ^14^ Milner Centre for Evolution University of Bath Bath UK; ^15^ Faculty of Biological Sciences Friedrich Schiller University Jena Germany; ^16^ Department of Human Evolutionary Biology Harvard University Cambridge Massachusetts USA; ^17^ Department of Anthropology Harvard University Cambridge Massachusetts USA

**Keywords:** ancient DNA, dental calculus, dental plaque, human microbiome, medieval, metagenomics, periodontal disease, *Tannerella forsythia*

## Abstract

**Objectives:**

Metagenomic investigations of ancient dental calculus provide insights into oral health, disease, and diet. Here, we analyze the dental calculus metagenome of King Richard III of England (1452–1485).

**Materials and Methods:**

Dental calculus DNA was extracted from three teeth of King Richard III and shotgun sequenced to a depth of nearly 400 million reads. The metagenomic data were taxonomically profiled and compared to new and previously published dental calculus metagenomes from England, Ireland, the Netherlands, and Germany spanning the Neolithic to the present. Sequencing data were *de novo* assembled, and metagenome‐assembled genomes assigned to the genus *Tannerella* were investigated for phylogenetic relatedness and virulence. Putative dietary DNA was assessed for authenticity.

**Results:**

The dental calculus of King Richard III was well‐preserved and yielded an exceptionally high quantity of DNA. Oral microbiome species diversity fell within the range previously observed among other northern European populations, suggesting that a royal lifestyle and a rich diet did not substantially impact his oral microbiota. The reconstructed *Tannerella* genomes contained many virulence factors found today among oral *Tannerella* species. No putative dietary DNA could be authenticated.

**Discussion:**

The dental calculus of King Richard III produced one of the richest ancient oral metagenomes published to date, yet the species diversity was indistinguishable from that of commoners living in northern Europe over the last 7000 years. Insufficient plant and animal DNA were recovered to investigate diet, suggesting that dental calculus may not be a sufficient source of dietary DNA even when exceptionally well‐preserved.

## 1 Introduction

In 2012, the skeletal remains of King Richard III of England were discovered beneath a car park on the grounds of the former Gray Friars church in the city of Leicester, UK (Buckley et al. 2013). Subsequent osteological (Buckley et al. 2013; Appleby et al. 2015; Appleby et al. 2014) and genetic and statistical (King et al. 2014) analyses of the evidence confirmed the identity of the remains of the long lost king. Richard III became king of England in 1483 during a period of civil conflict between the Plantagenet Houses of York and Lancaster later known as the Wars of the Roses (1455–1487). Remembered as a usurper, Richard III was accused by chronicler John Rous (Rous 1716) and Sir Thomas More (More 1883) of plotting the murder of his nephews to seize the throne. Crowned king at age 30, he reigned for two tumultuous years and was killed at the Battle of Bosworth in 1485, after which his body was hastily buried at the Gray Friars Church in Leicester. His life and brief reign became the subject of an eponymous tragic play by William Shakespeare in 1593 in which Richard III was portrayed as a ruthless villain (Shakespeare 1800). Although this literary legacy has made him one of England's most well‐known medieval kings, relatively little is known about his life other than its political outline, and the details of his rise to power following his brother's death remain sharply disputed (Baldwin 2015). Archaeology provides an opportunity to augment his scant historical record.

The discovery of Richard III's remains has provided an unusual chance to not only confirm specific historical details of his death but also to investigate less well‐understood aspects of his life. Analysis of his remains has confirmed his death in battle, as his skeleton bore the marks of at least 11 perimortem wounds, including two likely fatal wounds to the back of the head and several humiliation injuries that may have been inflicted after he was stripped of armor (Appleby et al. 2015). Historical accounts of a hasty burial have also been supported by the absence of evidence for a coffin, or even shrouding, with his corpse having been placed within a cramped grave, possibly with his hands bound (Buckley et al. 2013). Accounts of his uneven shoulder height are supported by the identification of idiopathic adolescent‐onset scoliosis in his skeleton (Appleby et al. 2014), which likely began during his training as a knight in Yorkshire. Well‐preserved DNA within his skeleton has confirmed his sex as male, and mitochondrial genome sequencing has conclusively linked him to two female‐line relatives (King et al. 2014). Isotopic analysis of his enamel bioapatite (Sr, Pb, O) and collagen from dentin and bone (O, C, N) has provided further insights into aspects of his life history (Lamb et al. 2014). His oxygen and strontium isotopic values are consistent with his childhood movements between eastern and western England, and his carbon and nitrogen isotopic values reflect a changing diet in childhood, followed by a likely increase in meat and high trophic level fish consumption from his teenage years onwards, and later a likely further increase in the regular consumption of luxury foods, such as wine, fish, and wildfowl, after being crowned king.

The excellent biomolecular preservation of the remains of Richard III suggests that an analysis of the DNA present in his dental calculus might yield further insights into his life, and specifically on his oral health and diet. Dental calculus, a mineralized form of dental plaque also known as tooth tartar, forms incrementally during life, entrapping the oral microbiota and serving as a long‐term biomolecular archive of the oral microbiome (Velsko and Warinner 2017; Warinner et al. 2015). Food biomolecules may also become entrapped in dental calculus and can further aid in dietary reconstruction (Warinner et al. 2014; Scott et al. 2021). However, while microbial metagenomics has been widely applied to reconstruct the microbial structure and diversity of the oral microbiome through time (Fellows Yates, Velsko, et al. 2021; Velsko and Warinner 2025) and to track the prevalence and virulence of periodontal pathobionts (Granehäll et al. 2021; Bravo‐Lopez et al. 2020; Philips et al. 2020; Jackson et al. 2024), the use of dental calculus metagenomics to study diet has been more limited (Gancz et al. 2023), and many challenges remain for authentication of DNA from dietary sources (Mann et al. 2023).

There is little evidence to date demonstrating that diet influences oral microbiome community structure. Many studies in living populations find no significant community‐wide differences (Galtier et al. 2026; Velsko, Fellows Yates, et al. 2019; Velsko et al. 2022; Sharma 2010; Bloch et al. 2019; Velsko et al. 2023), while changes in abundance of particular species may be study‐ or analysis‐specific (Kato et al. 2017). Ancient dental calculus sampled across major agricultural transitions likewise does not consistently reveal differences in oral microbial community structure pre‐ and post‐transition (Fellows Yates, Velsko, et al. 2021; Lokmer et al. 2020). While focused regional studies have attempted to identify oral microbiome changes in local populations corresponding to agricultural transitions, none have yet presented well‐supported evidence of a broader trend (Lassalle et al. 2018). The strongest evidence for dietary impact on oral microbiomes is between high sugar diets and elevated levels of species that ferment sugar to produce acids that erode tooth enamel, causing dental caries (Marsh 1994). Access to sugary foods was higher for medieval elite society than the general population, and a higher abundance of acid‐producing species such as *Streptococcus mutans*, implicated in caries development, might therefore be expected in the oral microbiome of King Richard III. However, *S. mutans* is rarely detected in ancient dental calculus, in part due to the dissolution of calculus in the presence of acid, and therefore tracing its historical impact may be challenging. This leaves us with an unclear understanding of whether and how social status may have historically impacted oral microbiota. To date, the majority of published metagenomic data from ancient dental calculus come from individuals who were not of high social status (Velsko, Fellows Yates, et al. 2019; Velsko et al. 2022; Eisenhofer et al. 2020) or whose social status is unknown. Analyzing the dental calculus metagenome of King Richard III offers the opportunity to investigate the oral microbiota and diet of a high‐status individual for which there are both historical records and archaeological data for comparison.

Phylogenetic reconstruction of microbial genomes from ancient dental calculus offers a unique opportunity to study the evolution of host‐associated microbes throughout human history and offers the opportunity to observe strain continuity across generations. Tracking genetic variation through time has revealed specific functional variation in methanogenic archaea (Granehäll et al. 2021), an evolutionary history of *Anaerolineaceae* bacterium oral taxon 439 that seems to track human migration (Ottoni et al. 2021), and an apparent recent gain of virulence genes in *Tannerella forsythia* (Jackson et al. 2024). To date, most studies have relied on mapping metagenome short reads to a pre‐selected reference genome to reconstruct ancient nucleotide diversity in a species of interest, yet alternative approaches offer additional genomic insights. For example, metagenome‐assembled genomes (MAGs) obtained through *de novo* metagenome assembly of ancient oral metagenomes revealed previously unrecognized oral methanogenic archaea (Granehäll et al. 2021), and enabled identification of species‐specific genes in both the known and novel archaeal species through a pangenome analysis. We recently used MAGs from diverse ancient and modern human populations to resolve cryptic diversity in the oral genus *Tannerella* (Galtier et al. 2026) highlighting the power of using metagenome assembly to clarify oral microbial diversity and genetic variation.

*Tannerella forsythia* is frequently investigated in ancient oral metagenomes due to its high prevalence and abundance in samples globally (Bravo‐Lopez et al. 2020; Philips et al. 2020; Jackson et al. 2024; Honap et al. 2023). This species is strongly associated with periodontal disease (Sharma 2010; Socransky et al. 1998), which is inflammation‐driven loss of gingival tissues and bone supporting the teeth, and therefore understanding the evolutionary history of *T. forsythia* is of interest to oral health research. However, the presence and abundance of the species are higher in historic dental calculus than in living individuals, and its relation to dental disease in historic populations is less clear (Velsko, Fellows Yates, et al. 2019; Velsko et al. 2022). Numerous virulence factors have been identified in *T. forsythia* through in vitro and in vivo investigation (Sharma 2010; Bloch et al. 2019), which provides a substantial background on which to investigate evolution of virulence in this species. Recovery of a *T. forsythia* MAG from Richard III presented the opportunity to investigate the phylogenetic placement and virulence repertoire of this species carried by a member of medieval elite society with evidence of generalized periodontitis across the dentition.

Here, we metagenomically characterize dental calculus from King Richard III and assess the microbial composition and presence of dietary DNA. This case study contributes to a growing body of literature investigating the health of the king and frames how medieval elite social status did not substantially shift oral microbial diversity compared to non‐elite individuals from locations in northern Europe spanning the last 7000 years. We found that the dental calculus of Richard III contains an extraordinary amount of well‐preserved DNA, and that the microbial species composition is indistinguishable from that of other historic‐era calculus from across northern Europe, suggesting that a royal lifestyle and a rich diet did not substantially alter his oral microbiota. We reconstructed metagenomically‐assembled genomes (MAGs) within the genus *Tannerella*, and found that the periopathogen *Tannerella forsythia* of Richard III falls within the known diversity and virulence of other modern and ancient *T. forsythia* in European populations. Putative dietary sequences could not be authenticated. Although deeper sequencing may provide additional dietary DNA sequences, the very low proportion of putative dietary sequences suggests that not all dental calculus may be a sufficient source of dietary DNA for dietary reconstruction, even when exceptionally well‐preserved.

## 2 Methods

### 2.1 Dental Calculus Sampling

Dental calculus samples of King Richard III were collected from lingual surfaces of three teeth: lower left first premolar (R31), upper left canine (R32), and lower right first premolar (R33). Prior to analysis, the dental calculus from the canine was split into two sub‐fractions, resulting in a total of 4 samples: R31 (GRF001.A), R32A (GRF001.B), R32B (GRF001.C), and R33 (GRF001.D) (Tables 1 and S1A). Six swabs from the archaeological storage facility were collected to monitor for possible contamination: sample bag (R3‐Bag), sample box (R3‐Box), storage refrigerator (R3‐Fr), laboratory gloves (R3‐Gl), osteologist hands (R3‐HS), and a laboratory water sample (R3MODBL) (Table S1A). Samples and swabs were transferred to the ancient DNA cleanroom facilities at the Laboratories of Molecular Anthropology and Microbiome Research (LMAMR) at the University of Oklahoma for DNA extraction.

**TABLE 1:** DNA extraction results.

Sample ID	Tooth	Calculus analyzed (mg)	Elution vol (μl)	DNA yield (ng/μL)	Normalized DNA yield (ng/mg)
R31	LP_1_	3.6	60	30.4	506.7
R32A	LC^1^	1.0	60	2.0	122.4
R32B	LC^1^	2.3	60	6.3	163.3
R33	RP_1_	4.0	60	25.8	387.0
R3‐AncBL1	—	—	60	^a^	—
R3‐AncBL2	—	—	60	0.2	—

*Note:* R3, Richard III.

^a^ Below detection.

### 2.2 Selection of Comparative Samples for Metagenomic Analysis

Published dental calculus metagenomic data sets from northern Europe dating 200–1000 BP were selected using AncientMetagenomeDir (accessed 2024/01) for (Fellows Yates, Andrades Valtueña, et al. 2021) (Table S3). These included samples from England (Velsko, Fellows Yates, et al. 2019; Fagernäs et al. 2020; Farrer et al. 2021), Ireland (Mann et al. 2018), the Netherlands (Velsko et al. 2022; Mann et al. 2018), and Germany (Warinner et al. 2014). The samples from England include 44 samples from the Radcliffe Infirmary, which served the general population of Oxfordshire in the mid‐eighteenth to mid‐nineteenth centuries (Velsko, Fellows Yates, et al. 2019), 29 samples from a medieval cemetery in York dated c. 800 BP (Weyrich et al. 2017; Farrer et al. 2021), and one additional sample from Yorkshire dated c. 700 BP (Fagernäs et al. 2020). The 37 samples from Ireland are from a single cemetery in Kilteasheen and are dated c. 1000 BP (Mann et al. 2018). The 78 samples from the Netherlands are from a single cemetery in Middenbeemster used by the general population in the mid‐eighteenth to mid‐nineteenth centuries (Velsko, Fellows Yates, et al. 2019). The two samples from Germany are from a medieval monastery in Dalheim dated c. 900 BP. Individual G12 of this data set is believed to have been a religious woman who illuminated manuscripts, based on the presence of lapis lazuli particles in her dental calculus (Radini et al. 2019). We additionally wished to compare the oral microbiome of Richard III to older samples, but there are no published well‐preserved calculus samples from Neolithic Britain and Ireland, so we instead chose to compare the exceptionally well‐preserved samples from Neolithic Germany. Dental calculus of three individuals from the Neolithic Linear b and keramik (LBK, 6th millennium BCE) site of Stuttgart‐Mühlhausen (Viesenhäuser Hof) in Baden–Württemberg, Germany (Price et al. 2003; Cox et al. 2024; Ash et al. 2016; Rivollat et al. 2020) were selected for analysis (Table S1A): gr. I‐064/335 (SMH005.A), gr. I‐056/528 (SMH007.A), gr. II‐027/274 (SMH008.A). Samples were transferred to the ancient DNA cleanroom facilities at the University of Tübingen, Germany, for DNA extraction.

### 2.3 DNA Extraction

DNA from the dental calculus of Richard III was extracted in a dedicated ancient DNA facility at LMAMR in accordance with established contamination control precautions and workflows. Two cleanroom non‐template extraction controls (R3‐AncBL1, R3‐AncBL2) were processed alongside the experimental samples during all analytical steps to monitor for possible contamination. Prior to decalcification, dental calculus samples were UV‐irradiated for 2 min using a Stratagene Stratalinker UV 1800 Crosslinker. To remove remaining surface debris and contaminants, they were agitated in 1 mL of 0.5 M EDTA solution for 15 min and decanted. The dental calculus samples were then processed using the DNA extraction protocol described in (Dabney et al. 2013), with minor modifications. In brief, the samples were crushed to powder using a sterile steel spatula and resuspended in 1 mL 0.5 M EDTA (Sigma) and incubated overnight at room temperature. A 100 μL proteinase K solution (> 600 mAU ml^−1^; Qiagen) was then added and incubated at 37°C for 8 h, followed by continued digestion under agitation at room temperature until decalcification was complete. The DNA was then purified using a MinElute column (Qiagen) connected to a Zymo reservoir (Zymo Research) following modified manufacturer instructions in which the amount of PB binding buffer was increased to 13 mL (Dabney et al. 2013). The DNA was eluted in 60 μL EB buffer (Qiagen), and the concentration of the eluate was quantified using 1 μL with a Qubit HS assay (Life Technologies).

DNA from the Stuttgart–Mühlhausen dental calculus samples was extracted in a dedicated ancient DNA facility at the University of Tübingen in accordance with established contamination control precautions and workflows, and following the DNA extraction protocol described in (Dabney et al. 2013) without modification.

### 2.4 Illumina Library Construction

Shotgun Illumina libraries (Table S1B) were constructed from 60 to 100 ng DNA from Richard III calculus samples and 30 μL of extract for non‐template extraction controls using a NEBNext DNA Library Prep Master set for 454 (New England Biolabs, E6070) according to manufacturer instructions. End repair was performed in 50 μL reactions with 100 ng of DNA. The end repair cocktail was incubated for 20 min at 12°C and 15 min at 37°C and then purified using a MinElute column (Qiagen) and eluted in 30 μL EB buffer. Blunt end adapters (IS1/IS3 and IS2/IS3) were prepared following Meyer and Kircher (2010) and ligated to the end‐repaired DNA in 50 μL reactions. The reaction was incubated for 15 min at 20°C and then purified using QiaQuick columns (Qiagen) and eluted in 30 μL EB. The adapter fill‐in reaction was performed in a final volume of 50 μL and incubated for 20 min at 37°C followed by 20 min at 80°C to inactivate the Bst polymerase. Libraries were amplified and indexed in a 50 μL PCR reaction, using 26.3 μL H_2_O, 5 μL 10× Platinum Taq MasterMix, 3 μL 2 mM dNTPs, 1 μL BSA (2.5 mg/mL), 1.5 μL 50 mM MgCl2, 1.5 μL i5 indexing primer (10 μM), 1.5 μL i7 indexing primer (10 μM), 0.2 μL PlatinumTaq, and 10 μL of library template. Thermocycling conditions were 2 min at 95°C, followed by 12 cycles of 15 s at 95°C, 30 s at 60°C, and 30 s at 72°C, and a final 7 min elongation step at 72°C. The optimal number of PCR cycles for each sample was first confirmed by qPCR, and all negative controls were amplified for 20 cycles. All libraries were purified using a MinElute PCR Purification kit (Qiagen) following manufacturer instructions and quantified using a Bioanalyzer 2100 High Sensitivity DNA assay (Agilent).

Shotgun Illumina libraries (Table S1B) were prepared similarly for the Stuttgart–Mühlhausen dental calculus samples, with minor modifications as described in the Non‐UDG Treated Double‐Stranded Ancient DNA Library Preparation for Illumina Sequencing protocol available on protocols.io (dx.doi.org/10.17504/protocols.io.bakricv6). A second aliquot of extracted DNA was then additionally prepared following USER enzyme treatment in order to remove DNA damage, as described in the Full‐UDG Treated Double‐Stranded Ancient DNA Library Preparation for Illumina Sequencing protocol available on protocols.io (dx.doi.org/10.17504/protocols.io.bqbpmsmn).

### 2.5 DNA Sequencing

Richard III DNA libraries (Table S1B) were pooled in equimolar amounts and sequenced on an Illumina HiSeq 2000 instrument with PE 2 ×100bp chemistry in RapidRun mode at the Yale Center for Genome Analysis (YCGA). A total of 393,365,842 sequencing reads were generated: R31, 73,678,628; R32A, 104,804,438; R32B, 125,374,688; R33, 89,508,088.

At the MPI‐EVA, the Stuttgart–Mühlhausen non‐UDG treated libraries (SMH005.A0101, SMH007.A0101, SMH008.A0101; Table S1B) were sequenced on an Illumina NextSeq 500 with PE 2 × 75bp chemistry, and the full‐UDG libraries (SMH005.A0102, SMH007.A0102, SMH008.A0102; Table S1B) were sequenced on an Illumina HiSeq 4000 with PE 2 × 75bp chemistry. A total of 724,359,015 sequencing reads were generated: SMH005.A, 213,889,522; SMH007.A, 164,169,600; SMH008.A, 346,299,893.

To generate more medieval dental calculus sequencing data for comparative analysis, we also deeply sequenced four previously generated DNA libraries (Table S1B) of individual G12 (S5, S6, S7, S8) from the site of Dalheim, Germany (Warinner et al. 2014) on two flow cells of an Illumina HiSeq 2000 using PE 2×150bp chemistry at the Yale Center for Genome Analysis (YCGA). A total of 240,767,032 sequencing reads were generated: S5, 86,797,120; S6, 55,998,288; S7, 50,206,795; S8, 47,764,829.

### 2.6 Data Processing

Raw data were processed using the nf‐core/eager v2.4.4 pipeline (Fellows Yates, Lamnidis, et al. 2021), including adapter trimming, read quality‐filtering (min length 30 bp, quality 40), and merging with adapter‐removal, mapping against human genome hg19 with bwa aln v0.7.17‐r1188 (Li and Durbin 2009), and selecting all reads that did not map to the human genome into a single fasta file with samtools v1.17 (Danecek et al. 2021). These fasta files of non‐human reads were used for taxonomic profiling.

### 2.7 Published Comparative Samples

**FIGURE 1:**
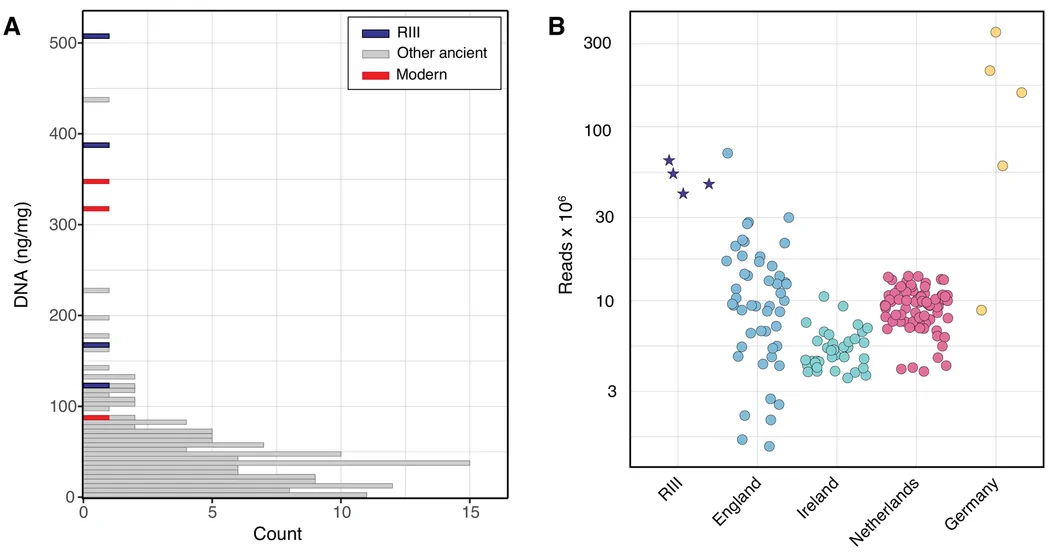
Quantification of extracted DNA and sequenced reads. (A) Normalized DNA recovery reported in ng DNA/mg dental calculus, with the dental calculus of Richard III (RIII) indicated in purple, other ancient dental calculus in gray, and modern dental calculus in red. (B) The number of DNA sequences per individual analyzed in this study (except the four libraries from Richard III which are reported separately), including both newly generated and published sequencing data after read processing and quality filtering; note use of log scale.

Normalized DNA extraction yields (ng DNA/mg calculus) measured in our laboratory from 143 archaeological dental calculus samples from diverse global sites and 3 present‐day individuals (Figure 1A) were obtained from the literature (Warinner et al. 2015; Warinner et al. 2014; Velsko et al. 2022; Mann et al. 2018; Velsko et al. 2024; Ziesemer et al. 2015; Ozga et al. 2016; Standeven et al. 2025) and are provided in Table S2.

Published dental calculus metagenomic data sets (Fellows Yates, Andrades Valtueña, et al. 2021) (Table S3, described above in “Selection of comparative samples for metagenomic analysis”) were downloaded from ENA and processed with the nf‐core/eager pipeline as described above for the new genetic data generated in this study. Data sets with fewer than 1M sequenced reads were excluded due to high stochasticity in identified species obfuscating signals of contamination or false‐positive identification vs. true presence. All negative controls and environmental swabs (R3‐AncBL1, R3‐AncBL2, R3Bag, R3Box, R3FR, R3GL, R3HS, R3MODBL) collected during sampling of the four calculus samples from Richard III fell below this threshold.

### 2.8 Taxonomic Profiling and Diversity Analysis

Newly sequenced and published metagenomic data sets ranged from < 3 M to > 300 M reads (Figure 1B), with most data sets between 5 M and 30 M reads. Notable exceptions were the newly sequenced libraries from King Richard III and from Germany (Stuttgart—Mühlhausen and Dalheim), which were intentionally deeply sequenced to facilitate *de novo* genome assembly. To improve data quality and consistency, samples with fewer than 5 M reads were excluded from all downstream analysis. All samples were taxonomically profiled with kraken2 v2.1.3 (Wood et al. 2019; Lu et al. 2022) using the GTDB v202 (Parks et al. 2022) database downloaded from the Struo2 (Youngblut and Ley 2021) ftp server (http://ftp.tue.mpg.de/ebio/projects/struo2/). Taxonomic tables were filtered in R v4.3.2 to remove potential contamination by removing all species with fewer than 5000 reads across all samples and then filtering out all species that were present at less than 0.01% relative abundance. The number of species and Shannon index were calculated in R using the package vegan v2.6–8 (Oksanen 2007). A principal component analysis (PCA) was run in R with the package mixOmics v6.26.0 (Rohart et al. 2017). After adding a value of +1 to all entries in the table to remove 0 values, a centered‐log ratio transformation (Gloor et al. 2017) was run on the data set and a PCA was performed on the transformed datatable. All diversity analysis results were plotted in R with ggplot2 (Wickham 2016).

### 2.9 Microbiome Preservation Assessment

Preservation of the samples was assessed using two methods. First, SourceTracker v1.0 (Knights et al. 2011) was applied to the newly generated data to determine whether the species profile was predominantly attributable to an oral source or to potential contamination sources (Table S4). Comparative sources included modern human dental calculus (Fellows Yates, Velsko, et al. 2021; Velsko, Fellows Yates, et al. 2019), dental plaque (Human Microbiome Project Consortium 2012), stool (Human Microbiome Project Consortium 2012; Obregon‐Tito et al. 2015; Rampelli et al. 2015), and skin (Human Microbiome Project Consortium 2012; Oh et al. 2016), as well as archaeological bone (Fellows Yates, Velsko, et al. 2021) and sediment (Slon et al. 2017). Second, the R package cuperdec (Fellows Yates, Velsko, et al. 2021) was used to assess the proportion of taxa from an oral source within all samples, including both newly generated sequences and published comparative data. Negative controls and one published dental calculus sample failed to meet the threshold for high oral content (Tables S2 and S3) and were excluded.

### 2.10 Dietary Analysis

For identification of putative dietary DNA sequences, we used the high‐throughput aligner MALT v0.4.0 (Vågene et al. 2018; Herbig et al. 2016) together with the NCBI nt database (October 2016) to taxonomically profile: the Richard III dental calculus samples (R31, R32A, R32B, and R33), negative controls (R3‐AncBL1, R3‐AncBL2), and environmental controls (R3Bag, R3Box, R3FR, R3GL, R3HS, R3MODBL); and the dental plaque (Human Microbiome Project Consortium 2012), stool (Human Microbiome Project Consortium 2012; Obregon‐Tito et al. 2015; Rampelli et al. 2015), skin (Human Microbiome Project Consortium 2012; Oh et al. 2016), archaeological bone (Fellows Yates, Velsko, et al. 2021) and archaeological sediment (Slon et al. 2017) data sets also used for preservation assessment. We employed a relaxed percent identity parameter of 85% and a base tail cut‐off (minimum support) of 0.01%. Resulting RMA6 files were loaded into MEGAN6 CE (Huson et al. 2016) and Operational Taxonomic Unit (OTU) tables were exported for Spermatophyta (seed plants) and Euteleostomi (major clade of vertebrates) (Table S5).

### 2.11 De Novo Genome Reconstruction

All metagenome samples were *de novo* assembled and binned using a Snakemake (Mölder et al. 2021) pipeline previously described in (Klapper et al. 2023), and which is available on GitHub under https://github.com/alexhbnr/ancient_metagenome_assembly. Assemblies were performed on an individual level, such that data sets from all libraries of a single individual were assembled together, resulting in a single output of metagenome‐assembled genomes from all four libraries of Richard III, as well as for G12. In brief, unmerged paired‐end sequencing data sets were assembled using MEGAHIT v1.2.9 (Li et al. 2015). After correcting the consensus contig sequences to remove miscoding lesions due to the presence of ancient DNA (Klapper et al. 2023), the contigs were binned using metaBAT v2.15 (Kang et al. 2019), MaxBin v2.2.7 (Wu et al. 2016), and CONCOCT v1.1.0 (Alneberg et al. 2014) and subsequently refined using metaWRAP v1.3.2 (Uritskiy et al. 2018). The resulting metagenome‐assembled genomes (MAGs) (Table S6) were further refined and validated and annotated with bakta v1.9.4 (Schwengers et al. 2021) using a Snakemake pipeline previously described in (Klapper et al. 2023) and available on GitHub under https://github.com/alexhbnr/automatic_MAG_refinement to remove potentially chimeric contigs.

### 2.12 Phylogenetic Analysis and Authentication

All genomes designated as *Tannerella forsythia* in NCBI databases were downloaded for phylogenetic comparison to the *de novo* reconstructed ancient genomes. The completeness and contamination of all reference genomes from NCBI were assessed with checkM v1.2.2 (Parks et al. 2015) using the program dRep v3.4.3 (Olm et al. 2017) (Table S6). A genome of *Tannerella serpentiformis* (GCA_003033925.1) was downloaded for use as an outgroup to root the tree. All NCBI genomes and all *de novo* reconstructed ancient genomes were used as input for the program PhyloPhlAn v3.1 (Segata et al. 2013; Asnicar et al. 2020) to reconstruct a tree based on selected marker genes specific for the species *T. forsythia* . Bootstrapping (200 replicates) was performed by RAxML using RAxML‐NG v1.2.2 (Kozlov et al. 2019) on the protein marker gene alignment file produced by PhyloPhlAn. The resulting tree was loaded into R and visualized with ggtree (Yu et al. 2017) with a midpoint root. Patristic distances were calculated from the tree using cophenetic.phylo in the R package ape v5.8 (Paradis et al. 2008) (Table S8).

The reconstructed genomes were confirmed to be of ancient origin by assessing the damage patterns on reads from each sample mapped against a *Tannerella* genome of the species selected based on the phylogenetic placement of the MAG produced from each species. This resulted in mapping to one of three species: *T. forsythia* reference genome (NC_016610.1), the Ca. *T. abscondita* genome with highest completeness (CMC011.B‐metaspades_076) (Galtier et al. 2026), or to Ca. *T. colubrina*, the *Tannerella serpentiformis*‐like species genome with highest completeness (GWHAPTL00000000.genome) (Zhu et al. 2021). All libraries were mapped using bwa aln v0.7.17‐r1188 with the flags ‐n 0.01 ‐o 2 ‐l 16,500. Mapped reads were filtered with samtools v1.17, and only reads with a mapping quality of ≥ 25 and a length ≥ 30 bp were considered suitable for damage analysis (Table S7). DamageProfiler v1.1 (Neukamm et al. 2021) was used to quantify DNA damage on mapped reads and calculate edit distance of mapped reads. Libraries of the sample G12 were additionally mapped against the *T. serpentiformis* reference genome (GCA_003033925.1), and the mapped reads were also filtered and analyzed using DamageProfiler.

### 2.13 Virulence Gene Content Assessment

The *Tanerella* genomes downloaded from NCBI were annotated with bakta (Schwengers et al. 2021) using the same settings as in the *de novo* assembly pipeline above. The annotated gene names were cross‐referenced against the known *T. forsythia* virulence factors listed in (Fellows Yates, Velsko, et al. 2021), and marked as 1 if present and 0 if absent (Table S6). The binary table was loaded into R and visualized as a matrix using tidyverse v1.3.0 (Wickham et al. 2019) and ggplot2 (Wickham 2016), and then combined with the phylogenetic tree using patchwork v1.2.0 (Pedersen 2017). The completeness and contamination of all genomes were likewise visualized in the same way using R.

## 3 Results

### 3.1 Exceptional DNA Recovery From the Dental Calculus of Richard III

We extracted DNA from four calculus samples collected from three teeth of Richard III (Table S1), and during laboratory processing we observed very high recovery of DNA, ranging from 122.4 to 506.7 ng/mg (Table 1). We compared this DNA recovery to 143 other archaeological dental calculus samples previously measured in our laboratories (Table S2), which ranged from 0.1 ng/mg to 437 ng/mg, with a median of 36 ng/mg (Figure 1A). We found that the amount of DNA recovered from the calculus of King Richard III is among the highest we have ever measured from an archaeological context, and is comparable to that at other recent archaeological sites with exceptional preservation in northern Europe (Dalheim, Tickhill, Middenbeemster) and Oceania (Rapa Nui). Such high DNA concentrations are similar to those measured from modern calculus (83.4–346 ng/mg) and suggest an excellent state of DNA preservation. To take advantage of this preservation and allow *de novo* genome assembly, we sequenced each dental calculus library from Richard III to a high depth totaling more than 400 million sequences (Figure 1B).

### 3.2 Well‐Preserved Oral Microbiota but no Confirmed Dietary DNA

**FIGURE 2:**
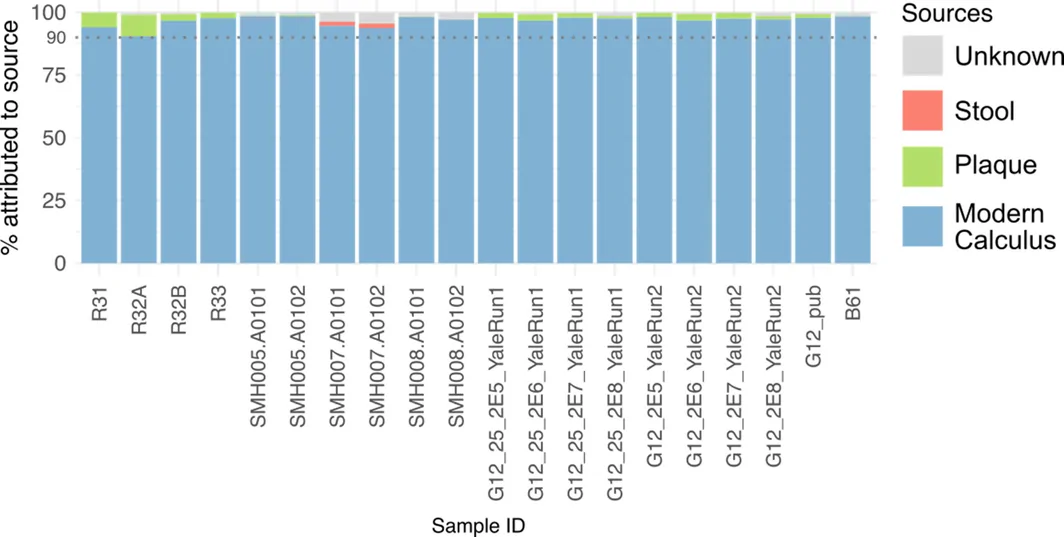
SourceTracker analysis of dental calculus preservation. All newly sequenced DNA libraries in this study exhibit a high preservation of oral microbial species with minimal contamination from other sources. Published data for sample G12 (G12_pub) and B61, from the same site, are shown for comparison. Dotted line indicates 90%. Sources that were included in SourceTracker analyses but were not detected in our samples (archaeological bone, sediment, and skin) are not shown in the plot.

Taxonomic classification of newly sequenced dental calculus from Richard III, Stuttgart–Mühlhausen, and the medieval site of Dalheim with Kraken2 (Wood et al. 2019; Lu et al. 2022) using the GTDB (Parks et al. 2022) indicated that all samples were exceptionally well‐preserved, with > 90% of DNA sequences attributed to an oral source (dental calculus or dental plaque) based on SourceTracker analysis (Figure 2; Table S4). Additional preservation assessment using cuperdec (Fellows Yates, Velsko, et al. 2021) confirmed a high proportion of oral taxa in these samples, as well as in the comparative dental calculus data sets (Figure S1).

Within the dental calculus of Richard III, however, no dietary sequences could be authenticated or confirmed. No plant sequences were identified following taxonomic classification with MALT (Vågene et al. 2018; Herbig et al. 2016) using the NCBI nt database, and non‐human animal sequences were limited to two species known to have contaminated and unreliable reference genomes (Mann et al. 2023): *Cyprinus carpio* (common carp, 4112 reads) and *Erinaceus europaeus* (European hedgehog, 42,683 reads) (Table S5). These two taxa were also identified in negative and environmental controls, further supporting their identification as false positives. Modern dental plaque likewise contained few dietary reads, with the largest number of reads assigned to contaminated genomes ( *Cyprinus carpio* ), model organisms (e.g., *Danio rerio* ), and wheat ( *Triticum aestivum* ) (Table S5). This suggests that dietary DNA incorporation and preservation within oral biofilms may be limited.

**FIGURE 3:**
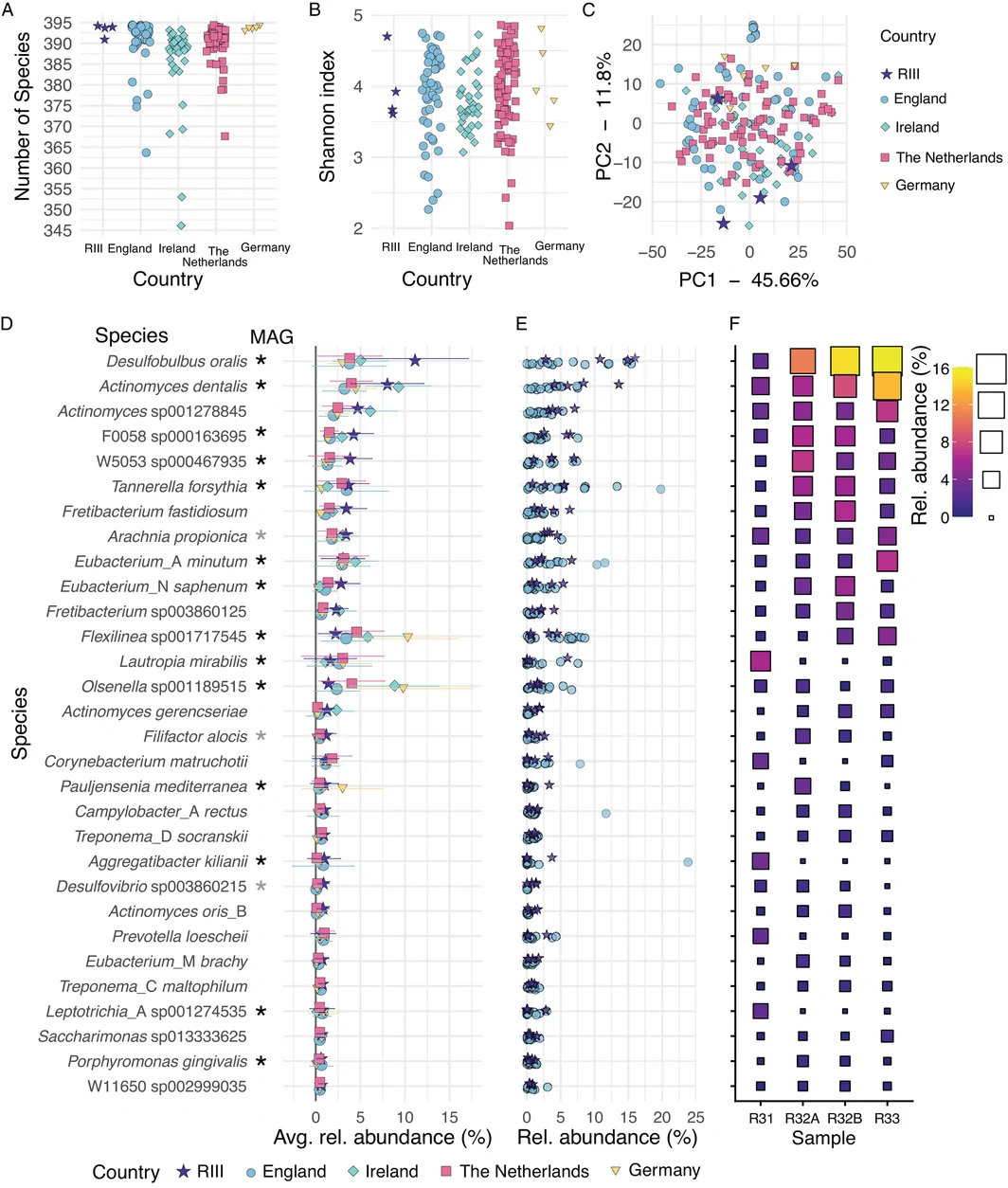
Microbiome metagenomic diversity analysis comparing calculus from King Richard III to other archaeological individuals in northern Europe. (A) Number of microbial species detected. (B) Shannon index of species evenness. (C) Principal component analysis (PCA) of between‐sample microbial diversity. (D) Average relative abundance of the 30 most abundant species in the Richard III samples, averaged per continent for comparative data, ranked from most to least abundant in the Richard III samples. An asterisk (*) next to the MAG indicates that a metagenome‐assembled genome (MAG) of this species was recovered from Richard III. A black *indicates that MAGs of this species were also recovered from comparative data; gray * indicates that no MAGs of this species were recovered from comparative data. (E) Per‐tooth relative abundance of the species in D for all samples from England sampled from incisors and canines. (F) Relative abundance of the species in D and E shown per tooth sampled from Richard III. Both box size and color correspond to relative abundance. The calculus sample from tooth R32 was split into 2 fractions for DNA extraction and sequencing, and the two fractions are indicated with A and B.

In contrast to dietary DNA, recovery of DNA from the oral microbiota was excellent. We identified nearly 400 microbial species in the dental calculus of Richard III, which is similar to the number of species detected in other well‐preserved archaeological dental calculus samples from England, Ireland, the Netherlands, and Germany (Figure 3A). Species evenness as measured by the Shannon index was similar across the dental calculus data sets (Figure 3B). Analysis of between‐sample diversity places the dental calculus of Richard III within the known microbial diversity of northern Europe (Figure 3C), indicating that the oral microbial profile of the king was not notably different from that of other individuals living in a broadly similar geographic region and time period. The main difference between the king's calculus and other archaeological dental calculus from northern Europe appears to be primarily the amount of microbial DNA preserved and not microbial ecological diversity.

We next investigated the most abundant species in dental calculus from Richard III and their distribution across our comparative data sets. The average relative abundance of the 30 most abundant species in the Richard III samples was calculated for all four Richard III samples together, and for the comparative data sets by continent (Figure 3D). Nine of the 10 most abundant species are notably higher in the Richard III samples; however, with only four samples and a high standard deviation, this may be an artifact of low sample counts. The abundant named species are well‐recognized oral taxa, and are anaerobic or facultatively anaerobic species commonly seen in ancient dental calculus. Several of these species are associated with periodontal disease including *Desulfobulbus oralis*, *Tannerella forsythia* , *Fretibacterium fastidiosum*, *Filifactor alocis* , and *Porphyromonas gingivalis* , and generalized periodontitis was observed across the dentition of Richard III. The 10 unnamed species, shown by a lack of a species name, are oral taxa with no formally recognized taxonomic species name (i.e., *Actinomyces* sp001278845 is *Actinomyces* sp. oral taxon 414), indicate that some abundant species in ancient dental calculus have not been the focus of culture‐based studies, and therefore their role in health and disease remains unknown.

To better understand how the relative abundance of these species in the Richard III samples compares to similar sample types, we investigated the relative abundance of these species per sample using only calculus samples from England (Figure 3E). When examined individually, the Richard III samples fall at the higher end of the abundance distribution of many species, yet are mostly within variation seen in other samples. We then plotted the abundance of each species per tooth (Figure 3F) to see how consistent species abundance is across dentition. There were notable differences in the relative abundance of several species on different teeth. The lower right first premolar, sample R33, had higher abundances of *Desulfobulbus oralis*, *Actinomyces dentalis* , *Eubacterium*_A *minutum*, and *Flexilinea sp001717545 (Anaerolineaceae* bacterium oral taxon 439) than the other teeth. The lower left first premolar, tooth R31, had higher relative abundance of *Lautropia mirabilis* , *Corynebacterium matruchotii* , *Aggretatibacter kilianii*, *Prevotella loescheii* , and *Leptotrichia*_A sp001274535 (*Pseudoleptotrichia* sp. HMT‐212), all of which were at the lowest levels of detection in the other teeth.

Both samples from the upper left canine, samples R32A and R32B, were consistent in their relative abundance of species, and had higher relative abundance of F0058 sp000163695 (*Bacteriodetes* oral taxon 274 strain F0058) W5053 sp000467935 (*Peptostreptococcaceae* bacterium oral taxon 113 strain W5053), *Tannerella forsythia* , *Fretibacterium fastidiosium*, and *Eubacterium*_N *saphenum* than either of the premolars. Generally, species relative abundance is more consistent between the two left teeth, R31 and R32, than between either left tooth and the right tooth R33. Although the calculus from Richard III had a high deposit mass, we find that many of the species that have a high relative abundance in Richard III's calculus were significantly enriched in samples with low deposit mass in an independent study, including *Filifactor alocis* , *Eubacterium*_N *saphenum*, and *Actinomyces gerencseriae* (Fagernäs et al. 2022). The reasons for this difference in enrichment remain unknown.

### 3.3 Phylogenetic and Virulence Factor Diversity of Tannerella forsythia

We performed *de novo* assembly and binning of our dental calculus metagenomes to examine evolutionary relationships of the species present in these samples without the bias of reference‐based mapping. This approach recovered metagenome‐assembled genomes (MAGs) of many of the species that were highly abundant in Richard III's calculus based on short read taxonomic profiling (Figure 3D column “MAG”). Very little laboratory work has been done to characterize most of these species, and few have multiple reference genomes in NCBI; therefore, linking evolutionary relationships within these species with human host behavior is challenging. We chose to focus on *Tannerella forsythia* because it is abundant, has multiple reference genomes, and its interactions with the host are relatively well‐studied.

*Tannerella forsythia* is a common oral species that is today associated with periodontal disease (Socransky et al. 1998), but which was historically, highly abundant in calculus on teeth with no evidence of disease (Velsko, Fellows Yates, et al. 2019). Although it was proposed that *T. forsythia* acquired virulence genes in the last thousand years (Jackson et al. 2024), we recently reported that these virulence gene‐lacking *Tannerella* represent the novel, previously unrecognized species Candidatus *Tannerella abscondita* (Galtier et al. 2026). We recovered one metagenome‐assembled genome (MAG) of *T. forsythia* from Richard III, four from the Radcliffe Infirmary in Oxford, England, and three from the Dutch site of Middenbeemster. We also recovered one genome each of Ca. *Tannerella abscondita* from the Radcliffe Infirmary Stuttgart–Mühlhausen in Germany, and one genome of Ca. *Tannerella colubrina* from Dalheim in Germany. One genome from the Radcliffe Infirmary could not be confidently placed in a species (Galtier et al. 2026). All 14 *Tannerella* genomes meet the minimum quality thresholds for medium and high‐quality MAGs (Bowers et al. 2017) used for present‐day genome reconstruction (Tables S6 and S7).

**FIGURE 4:**
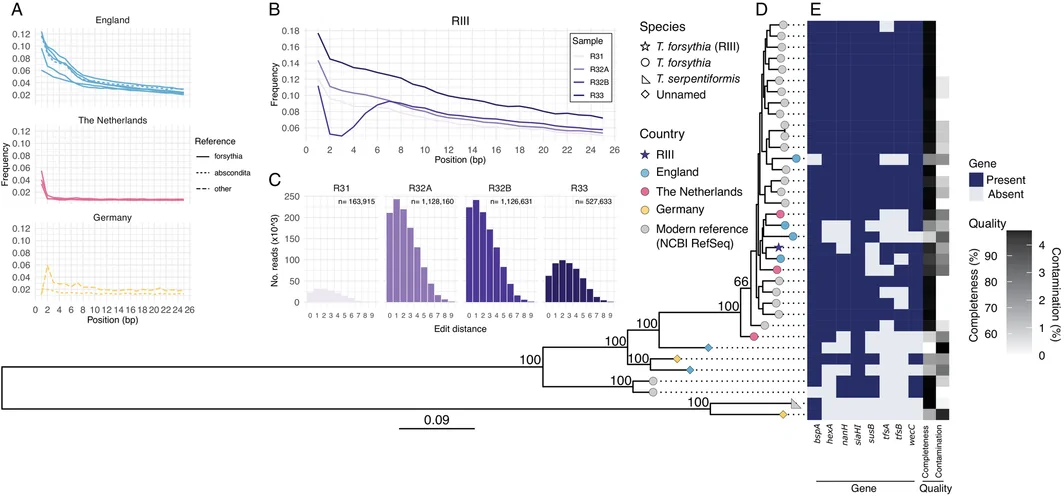
Analysis of ancient *Tannerella forsythia* phylogeny and virulence. (A) 5′ C‐to‐T damage patterns for each comparative dental calculus sample mapped against the *T. forsythia* reference genome (solid line), Ca. *T. abscondita* genome (dotted line), or Ca. *T. colubrina* genome (dashed line), grouped by country of origin. The reference species was selected based on the phylogenetic placement (panel D) of the MAG produced by each sample. Samples from England show expected damage patterns, while samples from the Netherlands were partial‐USER‐treated and show correspondingly low levels of damage on the first base. Minimal damage was observed for the German samples because they were processed using full‐USER treatment or a proofreading enzyme during library construction. (B) 5′ C‐to‐T damage patterns for each dental calculus sample from Richard III mapped against the *T. forsythia* reference genome. (C) Edit distance for reads mapped to the *T. forsythia* reference genome for each calculus sample from Richard III. The total number of reads mapped per sample (n) is shown in the top right corner of each panel. Edit distance distributions of R31 and R33 indicate that additional or alternative *Tannerella* species may be present in these samples. (D) PhyloPhlAn marker gene phylogeny of reference and ancient *de novo* reconstructed *Tannerella forsythia* genomes, mid‐point rooted. RAxML bootstrap values of deep nodes are shown. Recently proposed Candidatus species are marked as unnamed. (E) Presence–absence matrix of virulence factors for *T. forsythia* , as well as genome completeness and contamination, aligned with the tree tips in panel B. Genome labels are provided in Figure S2.

To confirm an ancient origin for the reconstructed *Tannerella* genomes, we mapped the reads from each individual (except the four libraries from Richard III which were each analyzed separately) that produced a *Tannerella* MAG against a *Tannerella* reference genome and assessed the 5′ C‐to‐T damage patterns (Figure 4A,B). The reference genome was selected based on the phylogenetic placement of the MAG produced by the sample being mapped (i.e., sample G12 produced a MAG that was phylogenetically placed in the Ca. *T. colubrina* clade, and was therefore mapped against a genome of this species). DNA sequences from Richard III and the Radcliffe Infirmary produced expected age‐related damage patterns, indicating that the *Tannerella* sequences in these samples originate from an ancient source (Briggs et al. 2007). Libraries from the Dutch site of Middenbeemster were prepared with partial‐USER treatment and showed an expected ancient DNA damage pattern of reduced damage only on the first base (Rohland et al. 2015). Libraries from the German sites were prepared with full‐USER treatment (Stuttgart–Mühlhausen) or a proofreading enzyme (Dalheim) and consequently show limited DNA damage (Briggs et al. 2010; Warinner et al. 2017). Notably, only the samples from the Netherlands show a baseline damage frequency of 0, while the others range between 0.025 and 0.075, suggesting that other *Tannerella* species may be present in these samples. This assessment is supported by the edit distance (number of mis‐matches) of the reads from each Richard III sample that mapped to the *T. forsythia* genome (Figure 4C).

In samples R32A and R32B, the most frequent edit distance value is 1, which indicates that *T. forsythia* is very likely the most abundant species present in these samples. Other *Tannerella* species may be present in lower abundance; hence, the high count of reads with 2 or more mis‐matches. In contrast, the maximum edit distance values in R31 and R33 is 2, and the curves are much broader, indicating that a higher percentage of reads had more than two mis‐matches in these two samples than in the canine. The number of reads mapped to the *T. forsythia* genome is much higher in R32A and R32B than R31 and R33, which is the same pattern we observed in taxonomic profiling (Figure 3F). Based on these results, it is possible that alternative *Tannerella* species may be present on different teeth and in lower relative abundances than is observed for *T. forsythia* . As only a single MAG each was recovered from assembly of Richard III and comparative samples, it is likely that additional *Tannerella* species are present but at lower abundance, and hence did not produce a MAG during assembly due to insufficient read counts. Until recently, only four *Tannerella* species were recorded as inhabiting the human oral cavity in the Human Oral Microbiome Database (eHOMD) v.4 (Chen et al. 2010; Escapa et al. 2018), and reference genomes were available for only two: *T. forsythia* and *T. serpentiformis*.

Recent phylogenetic analysis of metagenomically assembled *Tannerella* genomes has revealed that there are at least six different oral *Tannerella* species found in humans: *T. forsythia* group: *T. forsythia* , Ca. *T. abscondita* ; *T. serpentiformis* group: *T. serpentiformis*, Ca. *T. colubrina*, Ca. *T. anguiformis* , and Ca. *T. ophioides* (Galtier et al. 2026). Of these, three species have been recovered from ancient dental calculus: *T. forsythia* , Ca. *T. abscondita* , and Ca. *T. colubrina*. We built phylogenetic trees of the *Tannerella* MAGs recovered from our samples to explore the evolutionary relationships between them. Phylogenetic analysis of the *de novo* assembled genomes, along with reference genomes of *T. forsythia* from NCBI and the outgroup genome of *T. serpentiformis*, placed the ancient *T. forsythia* genomes from Richard III, England, and the Netherlands in a clade along with most of the reference genomes (Figure 4D). Two reference genomes that fall outside of the main *T. forsythia* clade were isolated from dogs and represent a distinct species (Galtier et al. 2026). Another two *Tannerella* genomes from England and Neolithic Germany fall outside of the main *T. forsythia* clade, and were recently confirmed to belong to the newly described Candidatus species Ca. *Tannerella abscondita* (Galtier et al. 2026). The last MAG from England that falls outside of the main *T. forsythia* clade is the least complete genome in our data set, and it is unclear whether it represents a separate species. Patristic distance calculations support these designations, as values within the *T. forsythia* clade are 0.053 when excluding these genomes, which is at the boundary of species‐level cut‐offs (0.05) (Velsko, Perez, and Richards 2019), while the average patristic distance within the clade when including genomes with deeper branches is 1.7, far higher than species‐level designations (Table S8). The final *de novo* assembled ancient genome from Germany falls within the *T. serpentiformis* group, within the species Candidatus *T. colubrina* (Galtier et al. 2026).

To assess the pathogenic potential of the reconstructed genomes in the broader *Tannerella* clade, we examined a selection of eight known virulence factors for *T. forsythia* (Figure 4E) using a previously curated list (Fellows Yates, Velsko, et al. 2021). The *T. forsythia* genome from Richard III is the only reconstructed MAG to include both surface layer (S‐layer) glycoprotein genes *tfsA* and *tfsB*, which may be because it is the most complete of the MAGs in this study. While these virulence factor genes are nearly ubiquitous in the reference genomes, several could not be detected in the *de novo* reconstructed genomes (Figure 4D), which may be due to the lower completeness of these genomes rather than a true absence (Galtier et al. 2026). For genomes that fall outside the main *T. forsythia* clade, a third or more of the virulence factor genes could not be detected, which corresponds to the reported differences in virulence gene repertoire between *T. forsythia* and other oral *Tannerella* species (Galtier et al. 2026). Overall, additional exploration of the genomic and genetic diversity of oral *Tannerella* is warranted to better understand how *de novo* metagenome assembly affects recovery of particular genes in *Tannerella*.

## 4 Discussion

We report the oral microbiome profile of four dental calculus samples from King Richard III of England, as well as new comparative dental calculus data from Neolithic and medieval Germany. DNA preservation within the dental calculus of Richard III was exceptional, but the reconstructed oral microbiota did not stand out from dental calculus of similar age and geographic origin regarding the number of microbial species detected, nor the abundance and distribution of microbial species. Despite the high DNA recovery and deep sequencing of the dental calculus metagenomes, no authentic dietary DNA was identified. Phylogenetic assessment of the abundant pathobiont *Tannerella forsythia* revealed that the *T. forsythia* genome reconstructed from Richard III is highly similar to genomes found in present‐day living populations, as well as in other archaeological dental calculus from Northern Europe.

We recovered an exceptionally high amount of DNA from the ancient dental calculus samples from Richard III, and Bayesian sourcetracking supported high DNA preservation from an oral microbial community. The metagenomic profile of the Richard III dental calculus did not stand out from comparative dental calculus from low‐status individuals, nor through time, and we were unable to identify any authentic dietary DNA, suggesting that exceptional preservation does not correspond to recovery of distinctive sources of nucleic acids. DNA extraction yield has not been extensively reported on in ancient dental calculus studies, yet more thorough explorations of the associations between the yield of extracted DNA and the amount of information that can be gleaned from it following sequencing are warranted. Our study was not designed to test associations with DNA density and recoverability of authentic ancient DNA compared to modern contamination, with the recoverability of non‐microbial DNA, or with microbial metrics of interest such as absolute abundance estimations, strain resolution, or recovery of low‐abundance species; however, exploring each of these may provide valuable insights into the limitations of current ancient DNA laboratory and sequencing protocols. Understanding how DNA preservation impacts the listed metrics may be useful for developing specific laboratory protocols designed to optimize recovery of nucleic acids derived from particular sources such as diet, or to produce data for targeted investigation of microbial abundances or strain delineation.

A wide range of dietary microfossils, proteins, and metabolites have been previously reported in modern and archaeological dental calculus (Scott et al. 2021; Velsko et al. 2017; Hendy et al. 2018; Radini and Nikita 2023), but dietary DNA is less well studied. Nevertheless, detection and authentication of dietary DNA in ancient dental calculus remains a potential source of information about diet at an individual level. While DNA sequences derived from particular meat and vegetable sources have been reported in ancient dental calculus (Warinner et al. 2014; Weyrich et al. 2017), few studies have reported on the presence or abundance of potential dietary reads, while those that have investigated dietary reads have generally been unsuccessful in authenticating them (Gancz et al. 2023; Mann et al. 2023; Velsko et al. 2024). In some cases, dietary DNA may be present, but the overwhelming abundance of microbial reads reduces the chances of dietary reads being incorporated into DNA libraries and sequenced. It is also possible that dietary DNA is mostly degraded by enzymes in saliva (Bradbury 1956) or in the microbial biofilm (Liu et al. 2017; Rostami et al. 2022), and very little is incorporated into dental calculus. Another possibility is that cooking breaks down DNA present in food items such that there is little DNA that might be incorporated, or cooking may fragment and expose dietary DNA making it highly susceptible to degradation by salivary nucleases.

Despite the deep sequencing of the dental calculus from Richard III, totaling nearly 400 million DNA sequences from three teeth, no authentic dietary DNA sequences were identified. Poor DNA preservation is unlikely to be the reason given the very high DNA recovery, which was similar to that from present‐day dental calculus, and the excellent preservation of the oral microbiome. Additional or deeper sequencing may eventually produce enough additional DNA sequences to identify potential dietary components, but this approach is expensive and not guaranteed to produce results. An alternative approach to recovering low‐abundance reads is targeted capture, which was shown to recover whole human mitochondrial genomes from ancient dental calculus (Ozga et al. 2016); however, this approach is limited by a limited number of reference genomes available for dietary plants and animals. Ultimately, in vivo models such as the one developed by Bartholdy, et al. (Bartholdy et al. 2025) are needed to test hypotheses regarding a lack of dietary read detection in ancient dental calculus. The low number of putative dietary sequences observed in present‐day dental plaque suggests that the incorporation and preservation of dietary DNA may be limited in oral biofilms. It is unclear why other dietary remains, such as plant microfossils and dietary proteins, are more robustly recovered from dental calculus, but at present it appears that dental calculus may not be a sufficient source of dietary DNA even when exceptionally well‐preserved and deeply sequenced.

Isotopic analysis of femur and rib fragments from the skeleton of King Richard III suggests that he consumed a diet rich in non‐local water sources, such as wine, and high trophic level game animals, such as fish and waterfowl, during the last 2–3 years of his life (Lamb et al. 2014). However, this aristocratic and unusually rich diet does not appear to have resulted in detectable microbial community changes compared to the dental calculus of lesser elites and commoners from across northern Europe, as all dental calculus analyzed in this study exhibited shared and overlapping species diversity. Whether the king's diet may have promoted the growth of specific individual oral microbial species, rather than affecting the entire microbial community, is unclear. To date, a direct relationship between diet and dental plaque biofilm species composition remains weakly and inconsistently supported in the literature (Wade 2021; Innocenti et al. 2023). Population‐level dental calculus data collected over time in a single region are needed to more finely track species and strain‐level microbial changes following a dietary change. Further advances in laboratory techniques that enable DNA extraction from distinct layers of microbial accumulation on dental calculus may also open the field to exploring oral microbiome changes throughout the lifetime of an individual.

The relative abundance of the most abundant species in Richard III's calculus varies across the three sampled teeth, yet falls mostly within the variation observed in comparative data sets. Many of the abundant species are anaerobic, late‐colonizer taxa that are more abundant in ancient dental calculus than in modern dental calculus or plaque, independent of dental disease (Velsko, Fellows Yates, et al. 2019). Despite the presence of generalized periodontal disease across Richard III's dentition, we cannot say which species we detected were actively involved in disease progression. Proteomic or metabolomic analyses of dental calculus (Velsko, Fellows Yates, et al. 2019; Velsko et al. 2017) may provide insight by revealing which species were actively producing known virulence factors.

The extent to which species abundance varies in calculus across the dentition in a single individual has not been extensively explored, although our results suggest that notable variation may be observed. Fagernäs, et al. (Fagernäs et al. 2022) performed DNA extraction and taxonomic profiling of ancient dental calculus across the dentition of four individuals and identified taxonomic variation between tooth surfaces (buccal, occlusal, lingual) and between teeth situated in anterior vs. posterior locations, although they did not report on tooth‐specific species abundance variation. Additional assessment of this data set revealed that abundances of species in the genus *Streptococcus* vary considerably across the dentition, in an apparently random manner (Velsko and Warinner 2025). The species abundance variation we observe across teeth from Richard III, and of *Streptococcus* species in other individuals (Velsko and Warinner 2025), may indicate that distinct communities develop on different tooth surfaces, which has been reported in dental plaque (Simón‐Soro et al. 2013; Proctor et al. 2018). The extent to which these tooth‐specific communities are consistent across individuals and through time, and whether different communities on corresponding tooth surfaces perform similar metabolic functions, remains to be investigated.

*Tannerella forsythia* is an oral bacterial species strongly associated with periodontal disease in living populations (Schäffer and Andrukhov 2024; Ready et al. 2008), and it is grouped together with *Porphyromonas gingivalis* and *Treponema denticola* within the “red complex” consortium of oral species with high pathogenic potential (Socransky et al. 1998). Our analysis of virulence genes within reconstructed *T. forsythia* genomes from ancient dental calculus found that these genes have been present in the species for at least several centuries, confirming earlier mapping‐based assessments (Bravo‐Lopez et al. 2020; Philips et al. 2020; Jackson et al. 2024). Although we did not detect all eight genes in each reconstructed genome, this absence could in part be an artifact of *de novo* metagenome assembly (Galtier et al. 2026). Current *de novo* assembly approaches rely on sufficient read length and coverage depth for genome reconstruction, and ancient DNA libraries may fall short of these lower limits (Borry et al. 2021).

Recent exploration of oral species diversity within the genus *Tannerella* revealed at least two previously undescribed human‐specific species, raising the number of potential *Tannerella* species in oral metagenomes from four to six, now each with an available genome or MAG (Galtier et al. 2026). In our phylogeny, 29 *Tannerella* genomes–including 3 historic Dutch genomes, 4 historic English genomes, and the genome from King Richard III–fall into a large clade that appears to represent true *T. forsythia* genomes. Only a single *Tannerella* genome from medieval Germany appears to belong to the recently named species Ca. *T. colubrina*, which is more closely related to *T. serpentiformis* than to *T. forsythia* (Galtier et al. 2026). However, one historic English and 1 Neolithic German *Tannerella* genome fall basal to the main *T. forsythia* clade, and are shown to represent Ca. *T. abscondita* .

In addition to confirming additional *Tannerella* species diversity, our data suggest higher *Tannerella* diversity within the dental calculus of specific individuals, and even within calculus on a single tooth surface. The elevated baseline damage frequency observed when mapping *Tannerella* DNA sequences from Richard III and other ancient individuals from England and Germany against their respective *Tannerella* reference genome supports that these calculus samples may contain more than one *Tannerella* species. The edit distance of reads from Richard III further supports this, as a high percentage of the reads have two or more mismatches, which indicates that the reads are from a different species than those against which they are mapped. Because only a single *Tannerella* genome was reconstructed from each sample, however, it is likely that one species is dominant and present at an abundance that enabled *de novo* genome assembly, whereas the other species are less abundant and did not have sufficient sequences for *de novo* assembly (Warinner et al. 2014; Borry et al. 2021).

The dental calculus of King Richard III is exceptionally well‐preserved and ancient DNA analysis has revealed an oral microbiome species composition that is highly similar to that of other human dental calculus from northern Europe. Despite excellent DNA preservation and deep sequencing of the DNA libraries, we were unable to identify authentic dietary DNA. We identified a higher level of taxonomic diversity in *de novo* reconstructed *Tannerella* genomes than has been reported to date in ancient dental calculus, indicating that further investigation of the diversity of the periodontal disease‐associated genus *Tannerella* is warranted to better understand how the oral microbiome has changed through human history.

## Author Contributions

**Alexander Hübner:** investigation, methodology, data curation, writing – review and editing, formal analysis, software. **Irina M. Velsko:** conceptualization, investigation, writing – original draft, methodology, validation, visualization, writing – review and editing, data curation, formal analysis, software. **Allison E. Mann:** investigation. **Courtney A. Hofman:** investigation. **Michael Francken:** resources. **Joachim Wahl:** resources. **Johannes Krause:** resources, funding acquisition. **Turi King:** conceptualization, writing – review and editing, project administration. **Anita Radini:** investigation. **Zandra Fagernäs:** investigation, writing – review and editing, formal analysis, software. **Camilla Speller:** investigation. **Andrew T. Ozga:** investigation. **Christina Warinner:** conceptualization, investigation, funding acquisition, writing – original draft, writing – review and editing, visualization, project administration, supervision, data curation, resources. **James A. Fellows Yates:** investigation, writing – review and editing, data curation, formal analysis, software. **Cecil M. Lewis Jr.:** funding acquisition, resources. **Sarah Fiddyment:** investigation.

## Funding

This study was supported by the Werner Siemens Stiftung (“Paleobiotechnology” to C.W.), the Deutsche Forschungsgemeinschaft (DFG, German Research Foundation) under Germany's Excellence Strategy (EXC 2051 Project‐ID 390713860, “Balance of the Microverse”), the Max Planck Harvard Research Center for the Archaeoscience of the Ancient Mediterranean (MHAAM), and the Max Planck Society.

## Ethics Statement

All required permissions were obtained to perform genetic data generation from newly analyzed archaeological dental calculus in this study. The excavation and analysis of the remains of Richard III was carried out by the University of Leicester Archaeological Services as part of the excavation of the Gray Friars Friary under a license granted by the U.K. Ministry of Justice. Permission to analyze remains from the site of Stuttgart–Mühlhausen was provided by the State Office for Monument Preservation in Konstanz, Germany.

## Conflicts of Interest

The authors declare no conflicts of interest.

## Supporting information


**Figure S1:** Cumulative percent decay (cuperdec) curves for ancient dental calculus samples including newly generated and published data as well as environmental controls (same as those used in SourceTracker) and extraction and library build blanks (Control). Samples are grouped by their original publication, titled by the first author's last name and the year of publication. Lines indicate the percent of all species (y‐axis) at and above a given abundance rank (x‐axis) that are classified as oral. Passed—False indicates the sample did not pass the preservation cut‐off, as defined by the modern dental calculus and other control samples. Passed—True indicates the sample passed the preservation cut‐off and is well‐preserved. The single sample that did not pass (from Farrer 2021) was excluded from all downstream analyses.
**Figure S2:** ajpa70350‐sup‐0001‐Figures.docx. *Tannerella forsythia* genome phylogenetic tree with tree tips labeled by genome.
**Figure S3:** 5′‐to‐3′ C‐ > T damage patterns on G12 libraries mapped against *Tannerella serpentiformis* or Ca. *Tannerella colubrina*. The number of mapped reads (*n*) is indicated under each reference name in the legend. Concatenated refers to all libraries, published and newly generated for this study, concatenated into a single fastq file for mapping. Non‐concatenated indicates individual libraries. The libraries for sample G12 were built with the Phusion enzyme, which is a proofreading enzyme and therefore the measured damage is low.

**Table S1.** Metadata for new samples and libraries in this study.
**Table S2.** Calculus DNA extract concentrations (ng/mg).
**Table S3.** Metadata for published datasets used in this study.
**Table S4.** SourceTracker results. For plotting, the proportions assigned to supPlaque and supPlaque were summed together as Plaque, and the proportions from ruralGut and urbanGut were summed together as Stool.
**Table S5.** MALT results for the Spermatophyta clade of seed plants and the Euteleostomi clade of vertebrate animals. The table includes RIII dental calculus, negative controls, and environmental controls, as well as comparative archaeological sediment, human stool, human skin, human supragingival plaque, and human subgingival plaque.
**Table S6.** Tannerella genome completeness, contamination, and virulence factor presence/absence. For viurlence factors, 1 indicates present and 0 indicates absent.
**Table S7.** Number of reads mapped against a reference genome, used as input for DamageProfiler.
**Table S8.** Patristic distance matrix for *Tannerella* phylogeny.

## Data Availability

All new sequencing data generated for this study were deposited in the European Nucleotide Archive under accession PRJEB84038 at https://www.ebi.ac.uk/ena/browser/view/PRJEB84038. Code used to perform analyses and generate the figures is provided in a GitHub repository at https://github.com/ivelsko/RIII_oral_micro.
